# Cortical Origin-Dependent Metabolic and Molecular Heterogeneity in Gliomas: Insights from ^18^F-FET PET

**DOI:** 10.3390/biomedicines13030657

**Published:** 2025-03-07

**Authors:** Huantong Diao, Xiaolong Wu, Xiaoran Li, Siheng Liu, Bingyang Shan, Ye Cheng, Jie Lu, Jie Tang

**Affiliations:** 1 Department of Neurosurgery, Xuanwu Hospital, Capital Medical University, Beijing 100053, China; 2 Department of Neurosurgery, China International Neuroscience Institute, Beijing 100053, China; 3 Department of Radiology and Nuclear Medicine, Xuanwu Hospital, Capital Medical University, Beijing 100053, China

**Keywords:** gliomas, ^18^F-FET PET, neocortex, mesocortex, molecular phenotyping

## Abstract

**Objectives**: The objective of this study is to explore the potential variations in metabolic activity across gliomas originating from distinct cortical regions, as assessed by O-(2-^18^F-fluoroethyl)-L-tyrosine positron emission tomography (^18^F-FET PET). Also, this study seeks to elucidate whether these metabolic disparities correlate with the molecular characteristics and clinical prognoses of the tumors. Specifically, this research aims to determine whether variations in ^18^F-FET PET uptake are indicative of underlying genetic or biochemical differences that could influence patients’ outcomes. **Methods**: The researchers retrospectively included 107 patients diagnosed with gliomas from neocortex and mesocortex, all of whom underwent hybrid PET/MR examinations, including ^18^F-FET PET and diffusion weighted imaging (DWI), prior to surgery. The mean and maximum tumor-to-background ratio (TBR) and apparent diffusion coefficient (ADC) values were calculated based on whole tumor volume segmentations. Comparisons of TBR, ADC values, and survival outcomes were performed to determine statistical differences between groups. **Results**: Among glioblastomas (GBMs, WHO grade 4) originating from the two cortical regions, there was a significant difference in the human Telomerase Reverse Transcriptase (*TERT*) promoter mutation rate, while no difference was observed in O^6^-Methylguanine-DNA Methyltransferase (*MGMT*) promoter methylation status. For WHO grade 3 gliomas, significant differences were found in the *TERT* promoter mutation rate and the proportion of 1p/19q co-deletion between the two cortical regions, whereas no difference was noted in *MGMT* methylation status. For WHO grade 2 gliomas, no molecular phenotypic differences were observed between the two cortical regions. In terms of survival, only GBMs originating from the mesocortex demonstrated significantly longer survival compared to those from the neocortex, while no statistically significant differences were found in survival for the other two groups. **Conclusions**: Gliomas originating from different cortical regions exhibit variations in metabolic activity, molecular phenotypes, and clinical outcomes.

## 1. Introduction

Gliomas are among the most common primary intracranial tumors. According to the World Health Organization (WHO) classification, gliomas are categorized into four grades (1–4) based on histological and molecular features [1]. Glioblastoma (GBM, WHO grade 4), in particular, is associated with a poor prognosis, with a median overall survival of only 14.6 to 20 months [2,3]. Gliomas can originate from various locations in the brain, with the frontal lobe being the most commonly affected region, followed by the temporal lobe and other brain areas [4]. Previous studies have demonstrated that tumor location is a critical prognostic factor, influencing tumorigenesis and tumor-specific genetic alterations. Supratentorial gliomas, for instance, exhibit worse overall survival compared to infratentorial gliomas and show significant differences in molecular and imaging biomarkers [4,5,6]. Recent findings further indicate that gliomas originating from different brain regions, such as the neocortex, mesocortex, and cerebellum, present distinct clinical profiles and molecular landscapes [4]. Notably, neocortical gliomas are more aggressive, with higher Ki67 indices and Telomerase Reverse Transcriptase (*TERT*) promoter mutation rates, compared to mesocortical gliomas, which exhibit longer survival and distinct molecular alterations, including lower Epidermal Growth Factor Receptor (*EGFR*) amplification and a higher prevalence of 1p/19q co-deletion [4].

O-(2-^18^F-fluoroethyl)-L-tyrosine positron emission tomography (^18^F-FET PET) has demonstrated significant advantages in metabolic imaging of gliomas [7,8]. Unlike conventional Contrast-Enhanced MRI (CE-MRI), it is not affected by the integrity of the blood–brain barrier and provides a more comprehensive depiction of metabolically active tumor regions [9]. This enables greater specificity and sensitivity in tumor grading, subtyping, and treatment target delineation. Hybrid imaging that combines ^18^F-FET PET with multi-parametric MRI has shown to improve the spatial delineation of tumor distribution, revealing metabolically active regions often exceeding those defined by MRI alone [9,10]. These regions have been validated through biopsy, emphasizing the potential of ^18^F-FET PET in guiding biopsy, gross total resection, and radiotherapy planning [9]. Moreover, ^18^F-FET uptake correlates strongly with molecular markers such as Ki67, reflecting tumor proliferation and heterogeneity. This makes it an invaluable tool in understanding glioma biology and optimizing individualized treatment strategies [9,10].

Despite advancements in glioma imaging, systematic investigations on the metabolic characteristics of gliomas originating from different cortical regions remain scarce. Moreover, it is unclear whether the metabolic features are linked to tumor molecular features and clinical outcomes. This study aims to provide new insights into their biological behavior and clinical management by elucidating the metabolic heterogeneity of gliomas from neocortex and mesocortex, integrating molecular characteristics and clinical outcomes.

## 2. Materials and Methods

### 2.1. Patients

**Figure 1:**
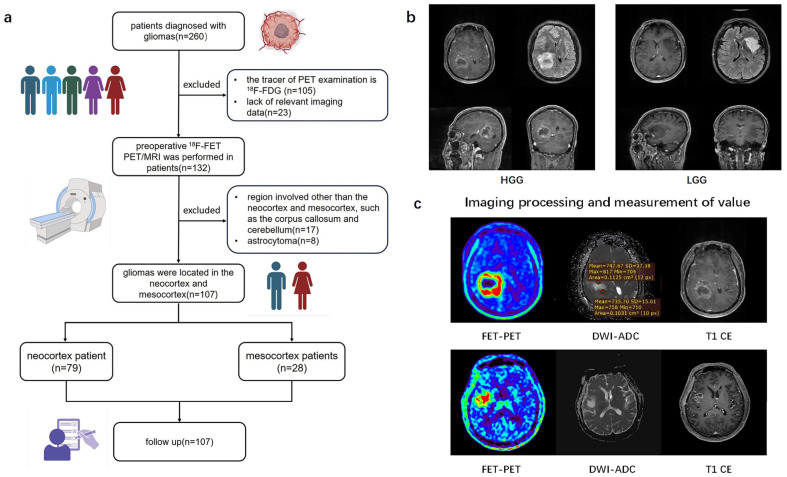
Study flow chart. (**a**) A flowchart of inclusion and exclusion criteria for glioma patients in the retrospective study. (**b**) Typical imaging of glioma originating from different cortical regions. (**c**) The value of TBR and ADC values measured from 3D ROI of tumors. ^18^F-FDG (2-[^18^F]-fluoro-2-deoxy-D-glucose); ^18^F-FET (O-(2-^18^F-fluoroethyl)-L-tyrosine positron emission tomography); HGG (high-grade glioma); LGG (low-grade glioma); DWI (diffusion weighted imaging); ADC (apparent diffusion coefficient); TBR (Tumor-to-brain ratio).

From July 2019 to August 2019, of the initial 260 patients, by the end a total of 107 patients with newly diagnosed or recurrent gliomas proven by pathology who underwent hybrid PET/MRI imaging prior to biopsy or surgical resection were retrospectively recruited into this study (Table 1). The study workflow is presented in Figure 1. Figure 1a shows the patient enrolment process. Based on brain MRI, gliomas involving the frontal, temporal, parietal, and occipital lobes are classified as the neocortex group, while those involving the cingulate gyrus and insular cortex are classified as the mesocortex group (Figure 1b). Written informed consent was obtained from all the patients before PET/MRI examinations. This study was conducted in accordance with the Declaration of Helsinki. This study was approved by the Medical Research Ethics Committee of Xuanwu Hospital, Capital Medical University. In accordance with the 2021 WHO classification, we collaborated with pathologists to reclassify glioma patients diagnosed prior to 2021 by integrating histopathological and molecular pathological results [1]. The inclusion criteria for this study were as follows: (1) adult-type diffuse gliomas pathologically diagnosed according to the 2021 WHO standards, (2) a tracer for PET examination of ^18^F-FET, (3) gliomas only involving the neocortex or mesocortex of the patient’s brain.

RNA sequencing data (Transcripts Per Million, TPM) correlated clinical information of patients with glioma (WHO grade 2–4) and were downloaded from TCGA (https://cancergenome.nih.gov/, accessed on 4 December 2024) database.

### 2.2. PET/MRI Imaging Acquisition and Reconstruction

All patients underwent integrated preoperative PET and MRI examinations using a 3-T time-of-flight (TOF) hybrid PET/MRI scanner (GE Signa, GE Healthcare, Chicago, IL, USA) equipped with a 19-channel head and neck coil. Accurate attenuation correction in PET/MRI scanning was based on MRI. A 3.0T brain MRI scan was performed to process the attenuation correction of PET images. The MRI sequences included a contrast-enhanced T1-weighted sequence (T1 CE), an axial T2-weighted (T2) sequence, a T2-weighted fluid-attenuated inversion recovery (T2 FLAIR; repetition time/echo time = 9000/maximum) sequence, and an axial diffusion-weighted imaging (DWI) sequence. All patients were required to fast for at least 4 h prior to intravenous injection of ^18^F-FET at a dose recommended for adult brain imaging (185–200 MBq). Image acquisition for FET-PET and multiparametric MRI scanning was performed simultaneously 20 min after the injection, with a total acquisition time of 20 min [11].

We used another 3.0-T PET/MRI hybrid scanner (uPMR790, United Imaging Healthcare, Shanghai, China) to conduct the PET image reconstruction. Image reconstruction utilized the TOF point-spread function-ordered subset expectation maximization (TOF-PSF-OSEM) method with four iterations and 20 subsets [12]. The parameters were as follows: matrix = 256 × 256, axial field of view = 30 cm, and slice thickness = 1.40 mm. The emission data were corrected for scatter, random effects, and dead-time coincidences [12].

### 2.3. Quantitative Analysis of Cerebral Metabolism and DWI

All images were post-processed and analyzed using MM BrainAnalysis software (version 2.1.3, United Imaging Intelligence, Shanghai, China). FET uptake was normalized using the standardized uptake value (SUV), which was calculated by dividing the radioactivity in the tissue (kBq/mL) by the injected radioactivity per gram of body weight. A banana-shaped or crescent-shaped volume of interest (VOI) was placed in the healthy cortex (gray and white matter) on the contralateral hemisphere of the lesion to serve as a reference for calculating the tumor-to-background ratio (TBR) (Figure 1c) [11]. Any voxels within the VOI with SUV exceeding the threshold due to vessels or other non-tumor structures were excluded. The biological tumor volume (BTV) was determined using a three-dimensional automatic contouring process with a TBR threshold of 1.6 or higher [13]. Mean Tumor-to-Background Ratio (TBRmean) was calculated by dividing the mean SUV of the tumor VOI by the mean SUV of normal brain tissue. Maximum Tumor-to-Background Ratio (TBRmax) was calculated by dividing the maximum SUV of the tumor VOI by the mean SUV of normal brain tissue. Apparent diffusion coefficient (ADC) maps were generated from DWI sequences. The ADC value for each tumor was obtained through region of interest (ROI) analysis, with ROIs carefully drawn to exclude areas of hemorrhage, cysts, and necrosis. The mean value of multiple ROIs was used to calculate the mean ADC (meanADC) (Figure 1c) [14].

### 2.4. Molecular Phenotypes

The included gliomas were pathologically diagnosed based on the 2021 World Health Organization (WHO) classification of central nervous system (CNS) tumors, incorporating histological subtypes, and relevant molecular markers. The mutation status of isocitrate dehydrogenase 1 (*IDH1*), Ki67 index, O^6^-Methylguanine-DNA Methyltransferase (*MGMT*) promoter methylation status, *TERT* promoter mutation status, and 1p/19q codeletion status were assessed using immunohistochemistry, Sanger sequencing, pyrosequencing, next-generation sequencing (NGS), and fluorescence in situ hybridization (FISH), respectively. Pathological results were jointly confirmed by two neuropathologists.

### 2.5. Statistical Analysis

Statistical analyses were performed using SPSS version 26 (IBM) and GraphPad Prism version 10.1.0. All quantitative data were expressed as mean ± standard deviation. The Shapiro–Wilk test was used to assess the normality of continuous variables. For continuous variables, Student’s *t* test or the Mann–Whitney U test (two-tailed) were used to evaluate statistical differences between groups. For categorical variables, Pearson’s chi-square test or Fisher’s exact test were applied. Descriptive statistics were presented as a mean with standard deviation or a median with range. To determine the accuracy of PET imaging in predicting *TERT* promoter mutation status, receiver operating characteristic (ROC) analysis was conducted. Imaging measurements were used as the diagnostic test, with histopathological analysis serving as the reference standard. The area under the ROC curve (AUC) and its 95% confidence interval were calculated. A *p*-value of less than 0.05 was considered statistically significant.

## 3. Results

### 3.1. Patient Enrollment and Tumor Characteristics

A total of 260 glioma patients who underwent preoperative integrated PET/MRI examinations were retrospectively reviewed at the beginning of this study. After excluding 153 patients, 107 eligible patients from July 2019 to August 2024 were included in the analysis. This cohort consisted of 64 males and 43 females, with a mean age of 51.7 ± 14.7 years (Table 1). In the neocortex group, there were 54 GBM patients, 12 WHO grade 3 and 13 WHO grade 2 glioma patients. Gliomas originating from the frontal lobe accounted for the highest proportion in this group (32 cases, 40.5%), followed by those from the temporal lobe (25 cases, 31.6%), parietal lobe (12 cases, 15.2%), and occipital lobe (10 cases, 12.7%). In the mesocortex group, there were nine GBM patients, eight WHO grade 3 and eleven WHO grade 2 glioma patients. The majority of gliomas in this group originated from the insula (27 cases, 96.4%), with only a small proportion arising from the cingulate gyrus (1 case, 3.6%). The mean age of patients in the neocortex group was 53.3 years (SD: 14.0), while the mean age in the mesocortex group was 47.3 years (SD: 16.1) (Table 2). There were no significant differences in age or sex between the two groups (*p* > 0.05). However, a significant difference was observed in the tumor composition between the two groups (a higher proportion of high-grade gliomas in the neocortex group compared to the mesocortex group, *p* < 0.01) (Table 2). Therefore, we further divided gliomas involving the neocortex and mesocortex into three groups based on WHO grading and conducted statistical comparisons between groups of the same grade.

### 3.2. Metabolic Differences in GBM Originating from Different Cortexs

**Figure 2:**
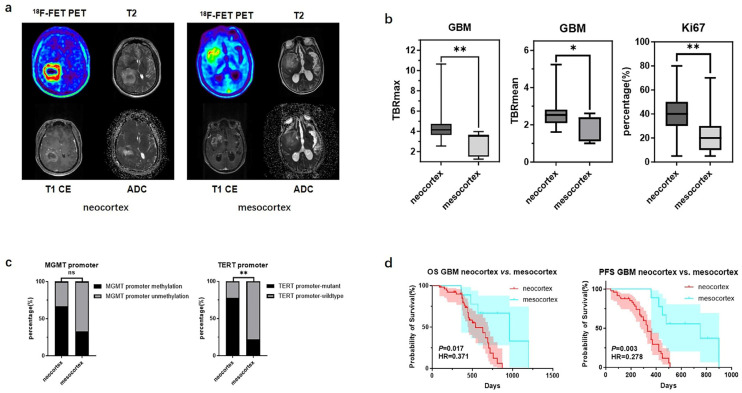
Mutation landscapes, clinical and imaging characteristics of GBMs originating from the neocortex and mesocortex. (**a**) The ^18^F-FET metabolic range of gliomas determined by semi-automatically segmenting the 3D ROI with a TBR > 1.6. (**b**) Comparison of TBRmax and TBRmean values in GBMs originating from different cortices (Student’s *t* test, *p* < 0.01, *p* < 0.05). Ki67 percentage in GBMs originating from the neocortex and mesocortex (Student’s *t* test, *p* < 0.01). (**c**) Comparison of mutation rates of key genes in GBMs originating from different cortices (Fisher’s exact test, *p* > 0.05, *p* < 0.05) (*, *p* < 0.05; **, *p* < 0.01; ns, not significant). (**d**) Comparison of OS and PFS between GBMs of neocortex and mesocortex origins (Kaplan Maier). ^18^F-FET PET, O-(2-^18^F-fluoroethyl)-L-tyrosine positron emission tomography; ADC, apparent diffusion coefficient; T1 CE, contrast-enhanced T1-weighted sequence; TBR, tumor-to-background ratio; TBRmax, maximum tumor-to-background ratio; TBRmean, mean tumor-to-background ratio; GBM, glioblastoma; OS, overall survival; PFS, progression-free survival; *TERT*, telomerase reverse transcriptase; *MGMT*, O^6^-Methylguanine-DNA Methyltransferase.

There were significant differences in TBR values and molecular testing results between GBMs originating from the two cortical regions. GBMs originating from the neocortex had higher TBRmax and TBRmean values compared to those from the mesocortex (4.46 ± 1.51 vs. 2.92 ± 1.12, *p* < 0.01; 2.57 ± 0.65 vs. 1.96 ± 0.67, *p* < 0.05) (Figure 2b) (Table 3). Additionally, the *TERT* promoter mutation rate and Ki67 index were significantly higher in neocortical GBMs (*TERT* promoter-mutant: 77.8% vs. 22.2%, *p* < 0.01; Ki67: 38.0% ± 15.5% vs. 20.9% ± 12.2%, *p* < 0.01) (Figure 2c) (Table 3). Meanwhile, we also observed that GBMs originating from the mesocortex had a better prognosis compared to those in the neocortex (Overall Survival (OS) 30 months vs. 18 months, *p* < 0.05; Progression-Free Survival (PFS) 25 months vs. 11 months *p* < 0.01) (Figure 2d) (Table 3).

### 3.3. Metabolic Differences in WHO Grade 3 and 2 Gliomas Originating from Different Cortexs

**Figure 3:**
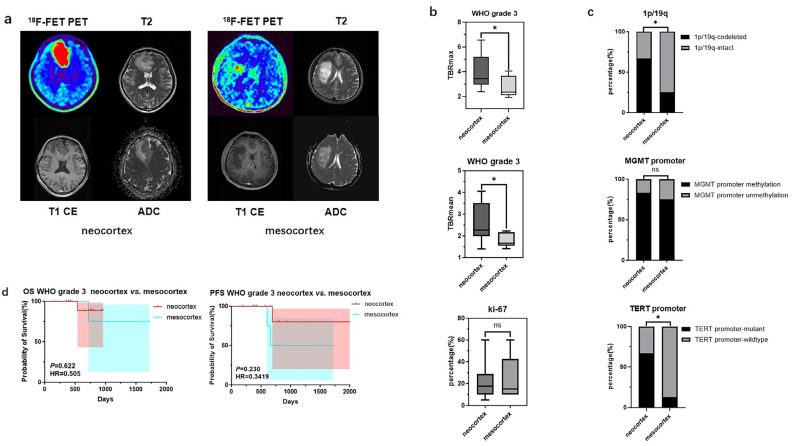
Mutation landscapes, clinical, and imaging characteristics of WHO grade 3 gliomas originating from the neocortex and mesocortex. (**a**) The ^18^F-FET metabolic range of gliomas determined by semi-automatically segmenting the 3D ROI with a TBR > 1.6. (**b**) Comparison of TBRmax and TBRmean values in WHO grade 3 gliomas originating from different cortices (Student’s *t* test, *p* < 0.05, *p* < 0.05). Ki67 percentage in WHO grade 3 gliomas originating from the neocortex and mesocortex (Student’s *t* test, *p* > 0.05). (**c**) Comparison of mutation rates of key genes in WHO grade 3 gliomas originating from different cortices (Fisher’s exact test, *p* < 0.05, *p* > 0.05, *p* < 0.05) (*, *p* < 0.05; ns, not significant). (**d**) Comparison of OS and PFS between WHO grade 3 gliomas of neocortex and mesocortex origins (Kaplan Maier). ^18^F-FET PET, O-(2-^18^F-fluoroethyl)-L-tyrosine positron emission tomography; ADC, apparent diffusion coefficient; T1 CE, contrast-enhanced T1-weighted sequence; TBR, tumor-to-background ratio; TBRmax, maximum tumor-to-background ratio; TBRmean, mean tumor-to-background ratio; OS, overall survival; PFS, progression-free survival; *TERT*, telomerase reverse transcriptase; *MGMT*, O^6^-Methylguanine-DNA Methyltransferase.

For WHO grade 3 gliomas, significant differences in TBR values and molecular testing results were also observed between samples originating from the neocortex and the mesocortex. The determination of glioma location and the processing of PET images can be seen in Figure 3a. WHO grade 3 gliomas from the neocortex had higher TBRmax and TBRmean values compared to those from the mesocortex (3.94 ± 1.39 vs. 2.72 ± 0.83, *p* < 0.05; 2.55 ± 0.88 vs. 1.79 ± 0.32 *p* < 0.05) (Figure 3b) (Table 4). Moreover, neocortical WHO grade 3 gliomas exhibited higher rates of *TERT* promoter mutation and 1p/19q co-deletion (*TERT* promoter-mutant: 66.7% vs. 12.5%, *p* < 0.05; 1p/19q-codeleted: 66.7% vs. 12.5%, *p* < 0.05) (Figure 3c) (Table 4). However, there was no statistically significant difference in prognosis between the two groups (Figure 3d).

**Figure 4:**
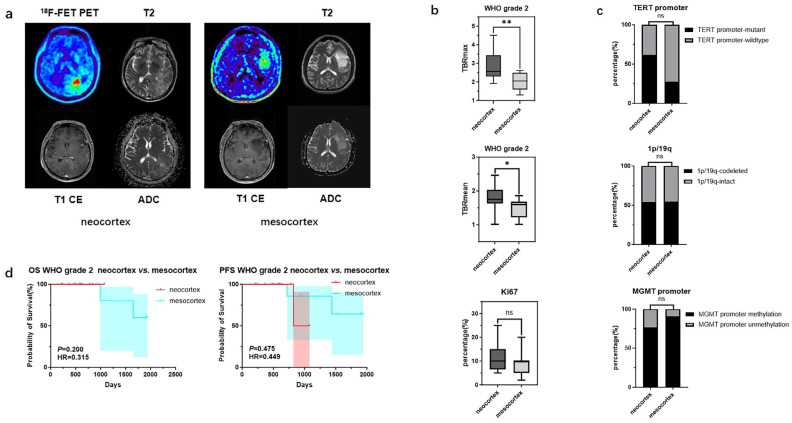
Mutation landscapes, clinical, and imaging characteristics of WHO grade 2 gliomas originating from the neocortex and mesocortex. (**a**) The ^18^F-FET metabolic range of gliomas determined by semi-automatically segmenting the 3D ROI with a TBR > 1.6. (**b**) Comparison of TBRmax and TBRmean values in WHO grade 2 gliomas originating from different cortices (Student’s *t* test, *p* < 0.01, *p* < 0.05). Ki67 percentage in WHO grade 2 gliomas originating from the neocortex and mesocortex (Student’s *t* test, *p* > 0.05). (**c**) Comparison of mutation rates of key genes in WHO grade 2 gliomas originating from different cortices (Fisher’s exact test, *p* > 0.05, *p* > 0.05, *p* > 0.05) (*, *p* < 0.05; **, *p* < 0.01; ns, not significant). (**d**) Comparison of OS and PFS between WHO grade 2 gliomas of neocortex and mesocortex origins (Kaplan Maier). ^18^F-FET PET, O-(2-^18^F-fluoroethyl)-L-tyrosine positron emission tomography; ADC, apparent diffusion coefficient; T1 CE, contrast-enhanced T1-weighted sequence; TBR, tumor-to-background ratio; TBRmax, maximum tumor-to-background ratio; TBRmean, mean tumor-to-background ratio; OS, overall survival; PFS, progression-free survival; *TERT*, telomerase reverse transcriptase; *MGMT*, O^6^-Methylguanine-DNA Methyltransferase.

In contrast, for WHO grade 2 gliomas originating from different cortical regions, differences were observed only in TBRmax and TBRmean values (2.86 ± 0.78 vs. 2.05 ± 0.50, *p* < 0.01; 1.78 ± 0.36 vs. 1.47 ± 0.30 *p* < 0.05), while no significant statistical differences were found in their molecular phenotypes and prognosis (Figure 4) (Table 5).

### 3.4. FET Uptake Levels and Prognosis Are Influenced by the Status of TERT Promoter and Ki67

**Figure 5:**
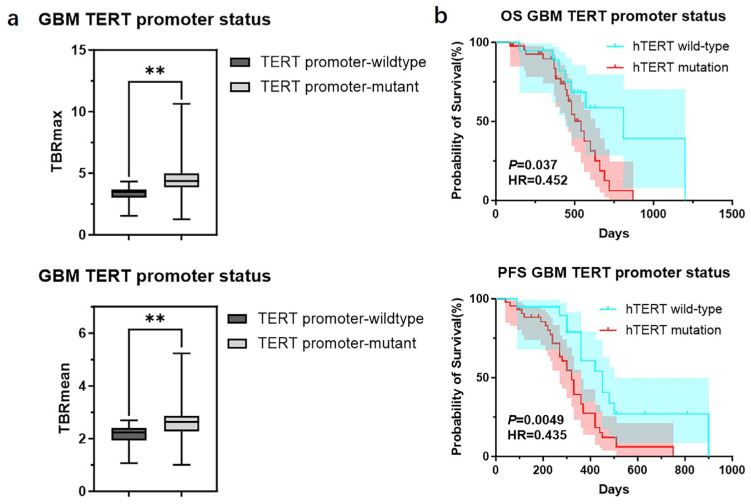
TBR values and clinical outcomes of GBMs divided into groups according to the status of *TERT* promoter. (**a**) Comparison of TBRmax and TBRmean values in GBMs with different *TERT* promoter status (Student’s *t* test, *p* < 0.01, *p* < 0.01) (**, *p* < 0.01). (**b**) Comparison of OS and PFS between GBMs with different *TERT* promoter status (Kaplan Maier). GBM, Glioblastoma; *TERT*, telomerase reverse transcriptase; TBR, tumor-to-background ratio; TBRmax, maximum tumor-to-background ratio; TBRmean, mean tumor-to-background ratio; OS, overall survival; PFS, progression-free survival.

**Figure 6:**
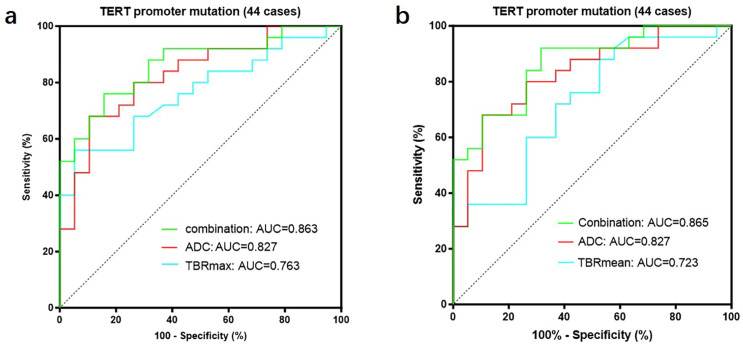
A total of 44 patients had both FET-PET and DWI-ADC imaging data. (**a**) The accuracy of predicting the TERT promoter status was evaluated using receiver operating characteristic (ROC) curves for TBRmax, ADC values, and their combination. The results showed that the area under the ROC curve was largest when both parameters were combined, indicating the highest accuracy (TBRmax: AUC = 0.763, ADC: AUC = 0.827, Conbination: AUC = 0.863). (**b**) The accuracy of predicting the TERT promoter status was evaluated using receiver operating characteristic (ROC) curves for TBRmean, ADC values, and their combination. The results showed that the area under the ROC curve was largest when both parameters were combined, indicating the highest accuracy (TBRmean: AUC = 0.723, ADC: AUC = 0.827, Conbination: AUC = 0.865). ROC curve for TBRmax, TBRmean and meanADC, in differentiating *TERT* promoter mutation from wildtype in gliomas. *TERT*, telomerase reverse transcriptase; TBR, tumor-to-background ratio; TBRmax, maximum tumor-to-background ratio; TBRmean, mean tumor-to-background ratio; ADC, apparent diffusion coefficient; AUC, area under the curve.

We further divided GBMs into two groups based on the status of the *TERT* promoter and compared the TBR values and prognosis between the two groups. The results showed that GBMs with *TERT* promoter mutations had higher TBR values (4.62 ± 1.65 vs. 3.39 ± 0.99 *p* < 0.01; 2.64 ± 0.75 vs. 2.12 ± 0.55 *p* < 0.01) and poorer prognosis (OS 18 months vs. 27 months *p* < 0.05; PFS 11 months vs. 15 months *p* < 0.05) compared to those with wild-type *TERT* promoters (Figure 5) (Table 6). Subsequently, we applied the same criteria to classify WHO grade 3 and WHO grade 2 gliomas. The results were consistent with those observed in glioblastomas, showing that gliomas with *TERT* promoter mutations exhibited higher metabolic activity than *TERT* wild-type gliomas (Supplementary Figure S2). Additionally, we conducted a correlation analysis between TBR values and Ki67 and found a positive correlation between them (Ki67 and TBRmax, R^2^ = 0.1318, *p* = 0.0001; Ki67 and TBRmean, R^2^ = 0.1482, *p* < 0.0001) (Supplementary Figure S3). Then, we used the receiver operating characteristic (ROC) curve for evaluation and obtained an area under the curve (AUC) of 0.763 (TBRmax) and 0.723 (TBRmean). Additionally, we found that the combination of TBR and ADC values yielded the highest AUC when predicting the status of the *TERT* promoter (TBRmax 0.763, meanADC 0.723, combination 0.863; TBRmean 0.723, meanADC 0.723, combination 0.865) (Figure 6).

We downloaded transcriptomic data of gliomas (WHO grade 2–4) from the TCGA database and divided the samples into two groups based on *TERT* promoter status. Comparing the two groups, we found that *SLC7A5* (solute carrier family 7 member 5) expression was higher in gliomas with *TERT* promoter mutations (Supplementary Figure S4).

### 3.5. Metabolic Differences Between Neocortical and Mesocortical Gliomas

Finally, we analyzed the metabolic and molecular phenotypic differences between neocortical and mesocortical gliomas without stratifying by tumor grade. There were significant differences in TBR values and molecular testing results between gliomas originating from the two cortical regions. Gliomas originating from the neocortex had a significantly higher mean TBRmax and TBRmean value compared to those from the mesocortex (4.17 ± 1.53 vs. 2.54 ± 0.93, *p* < 0.001; 2.44 ± 0.71 vs. 1.72 ± 0.49, *p* < 0.001). Moreover, the proportions of *IDH*1 and *TERT* promoter mutations were higher in neocortical gliomas than in mesocortical gliomas (67.9% vs. 31.6%, *p* < 0.01 and 68.4% vs. 25.0%, *p* < 0.001, respectively). In addition, the Ki67 index was significantly higher in neocortical gliomas compared to mesocortical gliomas (30.8% ± 18.0% vs. 17.6% ± 14.2%, *p* < 0.001). These findings suggest that gliomas originating from the neocortex exhibit significantly higher metabolic activity compared to those from the mesocortex (Supplementary Figure S1) (Supplementary Table S1).

## 4. Discussion

In this study, we analyzed 107 glioma cases treated at our institution, which originated from the neocortex and meso-cortex. All patients underwent pre-operative FET-PET/MRI imaging. Similarly to a previous study [4], gliomas originating from the neocortex and mesocortex may differ in terms of age at onset, with patients having neocortical gliomas tending to be older. However, no significant difference was observed, and future studies should focus on larger sample sizes. Grade heterogeneity of gliomas originating from the neocortex and mesocortex was found in the current study. Approximately 68.35% of gliomas from the neocortex and 32.14% of gliomas from the mesocortex were GBM. The percentage of GBM in gliomas of the neocortex is similar to the results of a previous study, which found that about 60% gliomas from the neocortex were GBM [4]. Additionally, Mackintosh et al. reported that 84% of gliomas in the temporal lobe were GBM [15].

Our study findings were the first to confirm that different neocortical gliomas (including GBM, WHO grade 3 and 2 glioma) exhibited significantly higher TBRmax and TBRmean values, indicating higher metabolic activity. Additionally, neocortical gliomas (including GBM, WHO grade 3 glioma) had significantly higher *TERT* promoter mutation rates and Ki67. Accordingly, survival analysis showed that neocortical GBMs had worse clinical outcomes, with shorter OS and PFS, suggesting greater proliferative potential and aggressiveness. Similar results could be found in a previous study [4], which reported that the *TERT* mutation rate in neocortical high-grade gliomas (WHO grade 3–4) was higher than that in mesocortical and the gliomas originating from the neocortex had significantly shorter OS and PFS compared to those from the mesocortex. Notably, this study is the first to reveal that gliomas of the same grade originating from the neocortex have significantly higher TBR values compared to those of the same grade originating from the meso-cortex. Although the TBR value alone cannot determine the exact malignancy of gliomas and requires a comprehensive evaluation incorporating pathological and molecular analyses, studies have shown that a higher TBR value is generally associated with a higher degree of malignancy in gliomas [8,9,16,17]. This correlation is mainly reflected in the fact that gliomas with higher TBR values often exhibit increased metabolic activity, greater proliferative and invasive potential, and are associated with higher-grade gliomas and poorer survival outcomes [16]. Specifically, gliomas with higher TBR values tend to have higher Ki67 and *TERT* promoter mutation rates, indicating increased tumor proliferative activity [9], this may be related to a higher number of active tumor cells, leading to increased FET uptake. As an imaging biomarker, the TBR value serves as an important reference for the comprehensive assessment of glioma malignancy and the formulation of treatment strategies. This study revealed significant clinical, metabolic, and molecular differences between gliomas originating from the neocortex and mesocortex.

In most tumors, including gliomas, *TERT* expression is associated with poor prognosis [18,19,20]. Typically, *TERT* promoter mutations are predictive of GBM survival and serve as an independent prognostic factor for poor outcomes [21]. Therefore, the worse clinical outcomes observed in GBMs originating from the neocortex compared to those from the mesocortex may be explained by the higher *TERT* promoter mutation rate. L-type amino acid transporter 1 (LAT1) is an essential neutral amino acid transporter that is highly expressed in tissues with high metabolic demands, including cancer cells [7,8,16]. Studies have shown that LAT1 is overexpressed in various malignant tumors, including GBM, and is closely associated with tumor invasiveness, proliferation, and treatment response [16]. Compared to normal brain tissue, LAT1 expression is significantly elevated in glioma tissues, with expression levels increasing in correlation with tumor grade, indicating a positive association with tumor malignancy. Furthermore, tumors with high LAT1 expression typically exhibit higher Ki67, reflecting enhanced cellular proliferative capacity [16]. The high expression of LAT1 is also associated with increased metabolic activity in ^18^F-FET-PET (^18^F-fluoroethyl-L-tyrosine PET) imaging, suggesting that LAT1 may play a crucial role in mediating amino acid uptake and promoting tumor cell metabolism and growth. Kaplan–Meier survival analysis reveals that patients with high LAT1 expression have significantly shorter OS and PFS compared to those with low LAT1 expression, further indicating that LAT1 could serve as a potential prognostic marker for poor outcomes in gliomas [7,8,16]. The high expression of LAT1 is usually accompanied by a high accumulation of FET in tumor tissue. This accumulation is reflected in PET imaging as higher TBR values [7,8]. Therefore, based on previous research findings [7,16], it may be inferred that the higher the expression level of LAT1, the higher the corresponding TBR values in gliomas. In this study, the TBR values of gliomas of the same grade originating from the neocortex were significantly higher than those from the mesocortex, which seems to suggest that the expression levels of LAT1 in neocortical gliomas are significantly higher than those in mesocortical gliomas. In this research, we found that gliomas originating from the neocortex have a higher *TERT* promoter mutation rate and higher TBR values compared to those originating from the mesocortex. Additionally, our analysis of glioma transcriptomic data from the TCGA database revealed that gliomas with *TERT* promoter mutations exhibit higher expression levels of SLC7A5. Since LAT1 is the protein encoded by the SLC7A5 gene [22], we hypothesize that the higher *TERT* promoter mutation rate in neocortical gliomas may lead to increased LAT1 expression, which in turn enhances FET uptake and consequently increases metabolic activity. The high expression of LAT1 in tumor cells and its differential expression in gliomas of different cortical origins may become a key focus for future research.

Our study has preliminarily found some differences in clinical outcomes and molecular phenotypes between gliomas originating from the neocortex and mesocortex. In particular, it was found that patients with glioblastoma originating from the mesocortex had a better prognosis than those with tumors arising from the neocortex. Several mechanistic hypotheses can be considered to explain this phenomenon: First, During the development of the cerebral cortex, the mesocortex develops earlier and consists of three to six layers, mainly responsible for processing emotions and feelings. In contrast, the neocortex develops later and consists of six layers, primarily responsible for higher cognitive functions [23,24]. The complexity of the neocortex implies greater plasticity in its cellular composition and a higher likelihood of mutations, which may contribute to more aggressive tumor characteristics [25,26,27,28]. Secondly, neurons and glial cells in the neocortex originate from the neuroepithelium and undergo complex migration and layering processes, whereas the mesocortex develops earlier and maintains a more conserved structure. The high plasticity and neural stem cell reservoir in the neocortex may provide tumors with greater adaptability for rapid progression and treatment resistance [27,28,29]. In addition, the neocortex has a richer vascular supply and higher metabolic demand, which may explain why gliomas originating from the neocortex exhibit higher TBR values on ^18^F-FET PET. This hypermetabolic state may enable tumor cells to grow more rapidly and invade more aggressively, potentially influencing their response to treatments such as radiotherapy and chemotherapy [25,29,30]. Finally, neurons in the neocortex form extensive connections across multiple brain regions, whereas the mesocortex has more restricted connectivity. Since gliomas tend to infiltrate along white matter fiber tracts, the widespread connections of the neocortex may provide tumor cells with more efficient pathways for dissemination, accelerating disease progression [25,26,31]. Although speculative, these hypotheses are based on the previous literature regarding the anatomy of the cerebral cortex, structure–function relationships, brain networks, energy metabolism, and neuron–glia interactions, making them appear credible. Therefore, we speculate that the differences in tissue structure and microenvironment between the neocortex and mesocortex during development may contribute to the differences in molecular mutations and clinical outcomes. In future studies, we will integrate genomics, proteomics, and metabolomics to further explore the molecular mechanisms underlying the metabolic activity differences between the neocortex and mesocortex. Additionally, we plan to expand the sample size and strive to conduct large-scale, multicenter studies to enhance the generalizability and clinical applicability of our findings. On this basis, we aim to develop more precise radiotherapy, chemotherapy, and targeted therapy protocols based on metabolic imaging and molecular characteristics.

## 5. Conclusions

Gliomas originating from the neocortex and mesocortex exhibit differences in clinical prognosis, cellular metabolism, and molecular phenotypes, which may assist neurosurgeons in preoperative planning and treatment decision-making.

## Figures and Tables

**Table 1:** Patient data.

Characteristic	Data
Total patients	107
Age	51.7 ± 14.7
Sex	
female	43 (40.2%)
male	64 (59.8%)
Neocortex	79 (73.8%)
frontal lobe	32 (29.9%)
temporal lobe	25 (23.4%)
parietal lobe	12 (11.2%)
occipital lobe	10 (9.3%)
Mesocortex	28 (26.2%)
insular lobe	27 (25.2%)
cingulate gyrus	1 (1.0%)

**Table 2:** Distribution.

	Neocortex	Mesocortex	*p* Value
Age	53.3 ± 14.0	47.3 ± 16.1	*p* > 0.05
Sex			*p* > 0.05
female	33 (41.8%)	10 (35.7%)	
male	46 (58.2%)	18 (64.3%)	
WHO grade			*p* < 0.01
GBM	54 (68.3%)	9 (32.1%)	
WHO grade 3	12 (15.2%)	8 (28.6%)	
WHO grade 2	13 (16.5%)	11 (39.3%)	

Student’s *t* test for comparing the difference in age between two cortices. Chi-square test for comparing the distribution of gender and tumor between two cortices.

**Table 3:** GBM neocortex vs. mesocortex.

Characteristic	Neocortex	Mesocortex	*p* Value
TBRmax	4.46 ± 1.51	2.92 ± 1.12	*p* < 0.01 ^1^
TBRmean	2.57 ± 0.65	1.96 ± 0.67	*p* < 0.05 ^1^
*TERT* promoter			*p* < 0.01 ^2^
mutant	42 (77.8%)	2 (22.2%)	
wildtype	12 (22.2%)	7 (77.8%)	
*MGMT* promoter			*p* > 0.05 ^2^
methylation	35 (64.8%)	3 (33.3%)	
unmethylation	19 (35.2%)	6 (66.7%)	
Ki67	38.0% ± 15.5%	20.9% ± 12.2%	*p* < 0.01 ^1^
OS, month	18	30	*p* < 0.05 ^3^
PFS, month	11	25	*p* < 0.01 ^3^

^1^ Student’s *t* test; ^2^ Fisher’s exact test; ^3^ Kaplan Maier. TBR, tumor-to-background ratio; TBRmax, maximum tumor-to-background ratio; TBRmean, mean tumor-to-background ratio; *TERT*, telomerase reverse transcriptase; *MGMT*, O^6^-Methylguanine-DNA Methyltransferase; OS, overall survival; PFS, progression-free survival.

**Table 4:** WHO grade 3 neocortex vs. mesocortex.

Characteristic	Neocortex	Mesocortex	*p* Value
TBRmax	3.94 ± 1.39	2.72 ± 0.83	*p* < 0.05 ^1^
TBRmean	2.55 ± 0.88	1.79 ± 0.32	*p* < 0.05 ^1^
*TERT* promoter			*p* < 0.05 ^2^
mutant	8 (66.7%)	1 (12.5%)	
wildtype	4 (33.3%)	7 (87.5%)	
*MGMT* promoter			*p* > 0.05 ^2^
methylation	10 (83.3%)	6 (75.0%)	
unmethylation	2 (16.7%)	2 (25.0%)	
1p/19q			*p* < 0.05 ^2^
codeleted	8 (66.7%)	1 (12.5%)	
intact	4 (33.3%)	7 (87.5%)	

^1^ Student’s *t* test; ^2^ Fisher’s exact test. TBR, tumor-to-background ratio; TBRmax, maximum tumor-to-background ratio; TBRmean, mean tumor-to-background ratio; *TERT*, telomerase reverse transcriptase; *MGMT*, O^6^-Methylguanine-DNA Methyltransferase.

**Table 5:** WHO grade 2 neocortex vs. mesocortex.

Characteristic	Neocortex	Mesocortex	*p* Value
TBRmax	2.86 ± 0.78	2.05 ± 0.50	*p* < 0.01 ^1^
TBRmean	1.78 ± 0.36	1.47 ± 0.30	*p* < 0.05 ^1^
*TERT* promoter			*p* > 0.05 ^2^
mutant	8(61.5%)	3(27.3%)	
wildtype	5(38.5%)	8(72.3%)	
*MGMT* promoter			*p* > 0.05 ^2^
methylation	10(76.9%)	10(90.9%)	
unmethylation	3(23.1%)	1(0.91%)	
1p/19q			*p* > 0.05 ^2^
codeleted	7(53.8%)	6(54.5%)	
intact	6(46.2%)	5(45.5%)	

^1^ Student’s *t* test; ^2^ Fisher’s exact test. TBR, tumor-to-background ratio; TBRmax, maximum tumor-to-background ratio; TBRmean, mean tumor-to-background ratio; *TERT*, telomerase reverse transcritase; *MGMT*, O^6^-Methylguanine-DNA Methyltransferase.

**Table 6:** *TERT* promoter status in GBM.

Status	*TERT* Promoter		*p* Value
	Mutation	Wildtype	
TBRmax	4.21 ± 1.65	2.97 ± 0.99	*p* < 0.01 ^1^
TBRmean	2.48 ± 0.75	1.91 ± 0.55	*p* < 0.01 ^1^
OS, month	18	27	*p* < 0.05 ^2^
PFS, month	11	15	*p* < 0.01 ^2^

^1^ Student’s *t* test; ^2^ Kaplan Maier; *TERT*, telomerase reverse transcriptase; GBM, glioblastoma; TBR, tumor-to-background ratio; TBRmax, maximum tumor-to-background ratio; TBRmean, mean tumor-to-background ratio; OS, overall survival; PFS, progression-free survival.

## Data Availability

Dates of this research are available from the corresponding author upon reasonable request.

## Supplementary Materials

The following supporting information can be downloaded at: https://www.mdpi.com/article/10.3390/biomedicines13030657/s1, Figure S1: TBR values of WHO grade 3 and 2 gliomas divided into groups according to the status of *TERT* promoter. (a). Comparison of TBRmax and TBRmean values in WHO grade 3 gilomas with different *TERT* promoter status (Student’s *t* test, *p* < 0.05, *p* < 0.05). (b) Comparison of TBRmax and TBRmean values in WHO grade 2 gilomas with different *TERT* promoter status (Student’s *t* test, *p* > 0.05, *p* < 0.01); Figure S2: Correlation analysis between TBR and Ki-67 (Simple linear regression, R^2^ = 0.1318, *p* = 0.0001; R^2^ = 0.1482, *p* < 0.0001); Figure S3: Comparison of SLC7A5 expression in gilomas with different *TERT* promoter status (Student’s *t* test, *p* < 0.05); Figure S4 Mutation landscapes, clinical and imaging characteristics of gliomas originating from the neocortex and mesocortex. (a) The 18F-FET metabolic range of gliomas determined by semi-automatically segmenting the 3D ROI with a TBR > 1.6. (b) Comparison of TBRmax and TBRmean values in gliomas originating from different cortices (Student’s *t* test, *p* < 0.0001, *p* < 0.0001). Ki-67 percentage in gliomas originating from the neocortex and mesocortex (Student’s *t* test, *p* < 0.001). (c) Comparison of mutation rates of key genes in gliomas originating from different cortices(chi-square test, *p* < 0.01, *p* > 0.05, *p* > 0.05, *p* < 0.001). (d) Comparison of OS and PFS between GBMs of neocortex and mesocortex origins (Kaplan Maier); Table S1: All neocortex vs. mesocortex.

## Abbreviations

The following abbreviations are used in this manuscript:

^18^F-FET PET	O-(2-^18^F-fluoroethyl)-L-tyrosine positron emission tomography
DWI	Diffusion weighted imaging
*TERT*	Telomerase Reverse Transcriptase
*MGMT*	O^6^-Methylguanine-DNA Methyltransferase
TBR	Tumor-to-brain ratio
ADC	Apparent diffusion coefficient
WHO	World Health Organization
GBM	Glioblastoma
EGFR	Epidermal growth factor receptor
TOF	Time-of-flight
T1 CE	Contrast-enhanced T1-weighted
T1 FLAIR	T1-weighted fluid-attenuated inversion recovery
T2 FLAIR	T2-weighted fluid-attenuated inversion recovery
SUV	Standardized uptake value
BTV	Biological tumor volume
VOI	Volume of interest
ROI	Region of interest
IDH	Isocitrate Dehydrogenase
NGS	next-generation sequencing
ROC	Receiver operating characteristic
OS	Overall survival
PFS	Progression-Free Survival
LAT1	L-type amino acid transporter 1
