# Supplementary material for: Cortical Origin-Dependent Metabolic and Molecular Heterogeneity in Gliomas: Insights from 18F-FET PET
Source: Biomedicines. 2025 Mar 7;13(3):657. doi: 10.3390/biomedicines13030657 (PMC11940755; doi:10.3390/biomedicines13030657)
Supplement: Supplementary file 1 [file biomedicines-13-00657-s001.zip › biomedicines-3476161-supplementary.pdf]

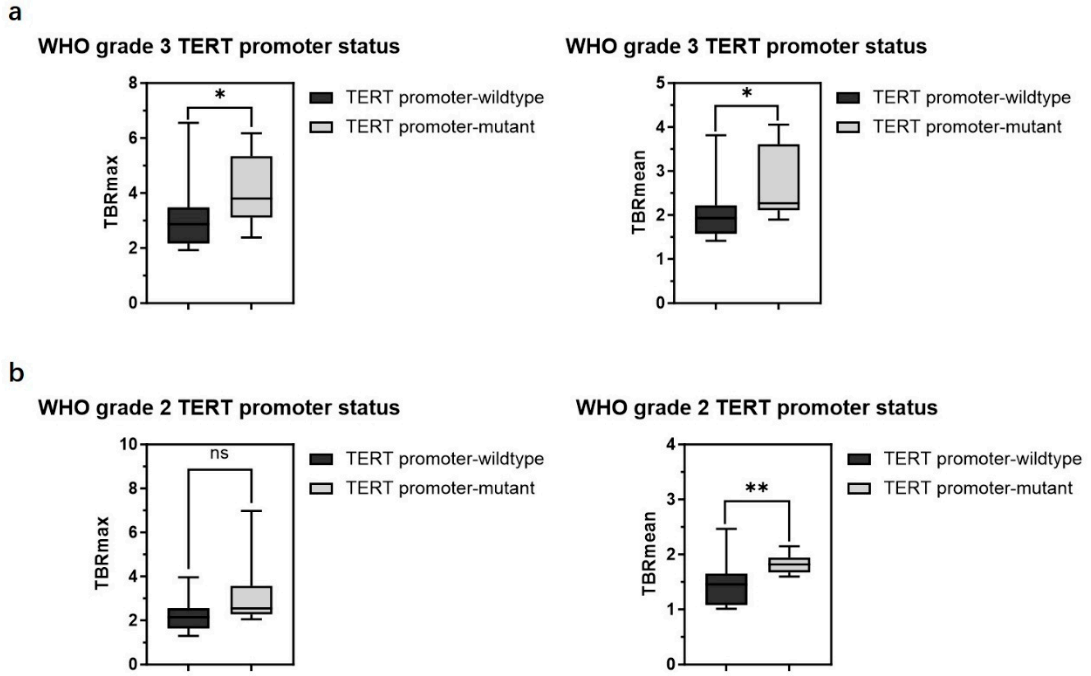

Supplementary Figure S1. TBR values of WHO grade3 and 2 gliomas divided into groups according to the status of *TERT* promoter. **a.** Comparison of TBRmax and TBRmean values in WHO grade 3 gilomas with different *TERT* promoter status (Student's t test, $p < 0.05$, $p < 0.05$). **b**. Comparison of TBRmax and TBRmean values in WHO grade 2 gilomas with different *TERT* promoter status (Student's t test, $p > 0.05$, $p < 0.01$). *TERT,* Telomerase Reverse Transcriptase; TBR, tumor-to-background ratio; TBRmax, Maximum Tumor-to-Background Ratio; TBRmean, Mean Tumor-to-Background Ratio.

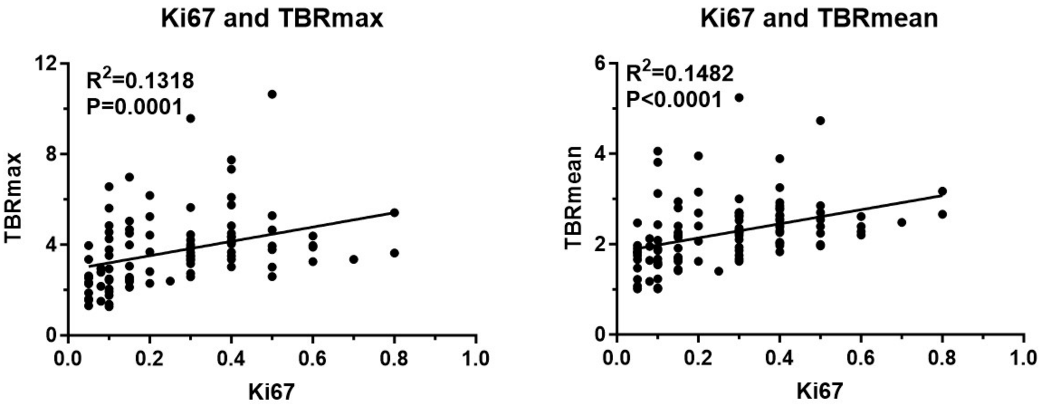

Supplementary Figure S2. Correlation analysis between TBR and Ki-67 (Simple linear regression, $R^2 = 0.1318$, $p = 0.0001$; $R^2 = 0.1482$, $p < 0.0001$). TBR, tumor-to-background ratio; TBRmax, Maximum Tumor-to-Background Ratio; TBRmean, Mean Tumor-to-Background Ratio.

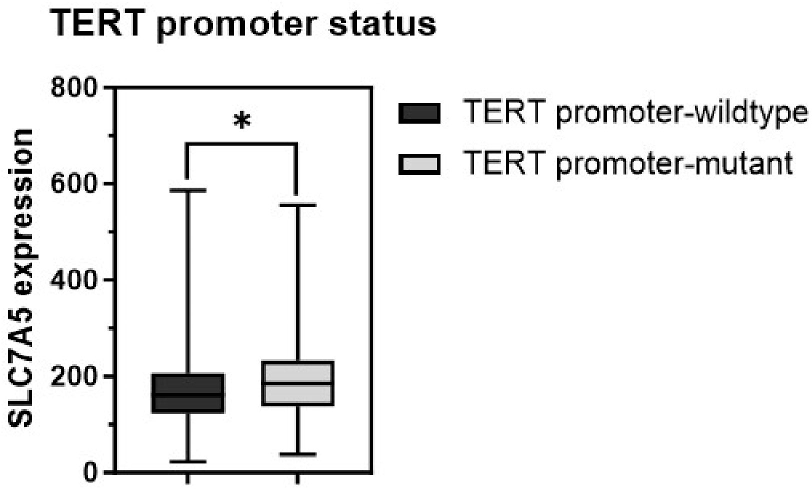

Supplementary Figure S3. Comparison of SLC7A5 expression in gilomas with different *TERT* promoter status (Student's *t* test, $p < 0.05$). *TERT,* Telomerase Reverse Transcriptase; SLC7A5, Solute Carrier Family 7 Member 5.

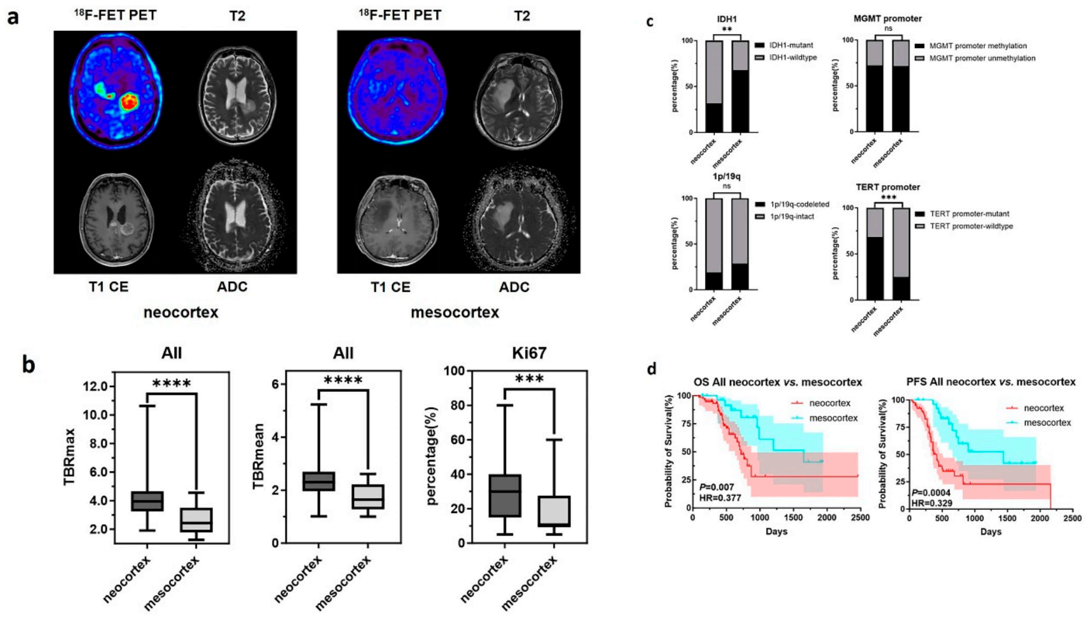

Supplementary Figure S4. Mutation landscapes, clinical and imaging characteristics of gliomas originating from the neocortex and mesocortex. a. The 18F-FET metabolic range of gliomas determined by semi-automatically segmenting the 3D ROI with a TBR>1.6. b. Comparison of TBRmax and TBRmean values in gliomas originating from different cortices(Student's *t* test, $p < 0.0001$, $p < 0.0001$). Ki-67 percentage in gliomas originating from the neocortex and mesocortex (Student's *t* test, $p < 0.001$). c. Comparison of mutation rates of key genes in gliomas originating from different cortices(chi-square test, $p < 0.01$, $p > 0.05$, $p > 0.05$, $p < 0.001$). d. Comparison of OS and PFS between GBMs of neocortex and mesocortex origins (Kaplan Maier). 18F-FET PET, O-(2-18F-fluoroethyl)-L-tyrosine positron emission tomography; ADC, apparent diffusion coefficient; T1 CE, contrast-enhanced T1-weighted sequence; TBR, tumor-to-background ratio; OS(overall survival); PFS(progression-free survival); TERT, Telomerase Reverse Transcriptase; MGMT, O6-Methylguanine-DNA Methyltransferase; IDH1, Isocitrate Dehydrogenase 1.

Supplementary Table S1. All neocortex vs. mesocortex

**Characteristic**	**Neocortex**	**Mesocortex**	***p* Value**
TBRmax	4.17±1.53	2.54±0.93	p < 0.00011
TBRmean	2.44±0.71	1.72±0.49	p < 0.00011
TERT promoter			p < 0.0012
mutant	54(68.4%)	7(25.0%)	
wildtype	25(31.6%)	21(75.0%)	
IDH1			p < 0.012
mutant	25(31.6%)	19(67.9%)	
wildtype	54(68.4%)	9(32.1%)	
MGMT promoter			p > 0.052
methylation	57(72.2%)	20(71.4%)	
unmethylation	22(27.8%)	8(28.6%)	
1p/19q			p > 0.052
codeleted	15(19.0%)	8(28.6%)	
intact	64(81.0%)	20(71.4%)	
Ki67	30.8%±18.0%	17.6%±14.2%	p < 0.0011
OS	24m	55m	p < 0.013
PFS	14m	48m	p < 0.0013

[1] Student's t test; [2] chi-square test; [3] Kaplan Maier. TBR, tumor-to-background ratio; TERT, Telomerase Reverse Transcriptase; MGMT, O6-Methylguanine-DNA Methyltransferase; IDH1, Isocitrate Dehydrogenase 1; OS, Overall Survival; PFS, Progression-Free Survival;
